# AAPM Task Group 298: Recommendations on certificate program/alternative pathway candidate education and training

**DOI:** 10.1002/acm2.13777

**Published:** 2022-09-20

**Authors:** Joann I. Prisciandaro, Jay W. Burmeister, Paul M. DeLuca, Bruce J. Gerbi, Maryellen L. Giger, James L. Robar, J. Anthony Seibert

**Affiliations:** ^1^ Department of Radiation Oncology University of Michigan Ann Arbor Michigan USA; ^2^ Karmanos Cancer Center, Department of Radiation Oncology Wayne State University School of Medicine Detroit Michigan USA; ^3^ Department of Medical Physics University of Wisconsin Madison Wisconsin USA; ^4^ Department of Radiation Oncology University of Minnesota Minneapolis Minnesota USA; ^5^ Department of Radiology University of Chicago Chicago Illinois USA; ^6^ Department of Radiation Oncology Dalhousie University Halifax Nova Scotia Canada; ^7^ Department of Radiology UC Davis Health Sacramento California USA

**Keywords:** education, medical physics, training

## Abstract

Entry into the field of clinical medical physics is most commonly accomplished through the completion of a Commission on Accreditation of Medical Physics Educational Programs (CAMPEP)‐accredited graduate and residency program. To allow a mechanism to bring valuable expertise from other disciplines into clinical practice in medical physics, an “alternative pathway” approach was also established. To ensure those trainees who have completed a doctoral degree in physics or a related discipline have the appropriate background and didactic training in medical physics, certificate programs and a CAMPEP‐accreditation process for these programs were initiated. However, medical physics–specific didactic, research, and clinical exposure of those entering medical physics residencies from these certificate programs is often comparatively modest when evaluated against individuals holding Master's and/or Doctoral degrees in CAMPEP‐accredited graduate programs. In 2016, the AAPM approved the formation of Task Group (TG) 298, “Alternative Pathway Candidate Education and Training.” The TG was charged with reviewing previous published recommendations for alternative pathway candidates and developing recommendations on the appropriate education and training of these candidates. This manuscript is a summary of the AAPM TG 298 report.

## 1 PREAMBLE

Medical physics is a profession requiring a broad skill set. Moreover, it has long been recognized that breadth in the educational background of those entering our profession enhances contributions to the medical community. However, the increasing complexity of clinical demands within the respective subfields of medical physics has led to additional emphasis placed on the standardization of the underlying training, for example, Paliwal et al.^1^ Therefore, a trade‐off must exist between the depth and breadth of training relative to the quality of standard practice and the capacity to innovate, respectively. An appropriate depth of training for graduates is fundamental to the successful practice of medical physics. Hence, the standardization of trainee background prior to medical physics graduate training results in more uniformity in the preparation of these graduates (e.g., see the Commission on Accreditation of Medical Physics Education Programs [CAMPEP] Standards for Accreditation of Graduate^2^ and Residency^3^ Educational Programs in Medical Physics). However, increased standardization carries a risk that we restrict our graduates to thinking “inside the box,” potentially limiting and compromising research and innovation. Furthermore, intellectual diversity in background training is beneficial due to the array of complex duties associated with our profession. Such diversity enhances our ability to discover, innovate, translate, and implement future advances in current practice domains as well as emerging domains.

The current CAMPEP accreditation standards for graduate training in medical physics requires a strong foundation in basic physics.^2^ Recently, there has been significant discussion of broadening the recommended preparation of entrants into medical physics education programs to enhance the role of the medical physicist in the clinical setting, as well as to prepare students for other potential career paths (e.g., academia, industry, and government). Current educational prerequisites for entrants into accredited graduate programs are provided in the CAMPEP accreditation standards.^2^ The “alternative pathway” into the profession, as described by Task Group (TG) 133^4^ and AAPM Report 197S,^5^ and modified by CAMPEP to only allow doctoral degree holders,^2^ has traditionally represented a mechanism for bringing valuable expertise from other disciplines into medical physics. The “certificate program” was envisioned as a means to formalize the medical physics didactic training for such entrants to ensure a common initial starting point and to allow graduates to succeed in residency training. AAPM Report 197S^5^ recommends six core topics as a minimum requirement for such programs. However, medical physics–specific didactic, research, and clinical exposure of those entering a medical physics residency from these certificate programs is often comparatively modest when evaluated against individuals holding Masters and/or Doctoral degrees in CAMPEP‐accredited medical physics programs. In addition, CAMPEP allows two core topics to be covered as remedial education during a medical physics residency,^3^ thus further lowering the required pre‐residency preparation of these entrants.

Since the approval of AAPM Report 197S^5^ and the subsequent implementation of certificate programs, a number of questions have arisen regarding the preparation of medical physics trainees within this alternative pathway as summarized in the following list:

- Have the educational requirements for alternative pathway candidate's entry into clinical training programs been adequately defined? That is, are the core elements specified in AAPM Report 197S sufficient?
- In what environment should certificate programs exist?
- Can a trainee gain adequate clinical exposure and professional understanding through training in a certificate program?
- If online training is acceptable, in which format should it be delivered (e.g., prerecorded lectures, virtual classrooms), and for which courses? As an example, this question was particularly relevant during the initial 6–12 months of the COVID‐19 pandemic.
- Should a trainee be permitted to concurrently complete a non‐CAMPEP‐accredited PhD and certificate program?
- How are we evaluating the potential success of the certificate program in formalizing the alternative pathway, in preparing alternative pathway candidates for entry into the profession, and, ultimately, in contributing to advancements in the science and clinical application of medical physics?
- Is this approach consistent with attracting the best possible scientists to our field?
- Should the alternative pathway be expanded, contracted, or remain at its current capacity?

AAPM TG 298, “TG on Alternative Pathway Candidate Education and Training,” was established to review previous recommendations for alternative pathway candidates and to provide recommendations on how to optimize the practical implementation of the alternative pathway in the interest of both our profession and potential future entrants into it.

## 2 INTRODUCTION

Although the benefits of preserving a diverse array of backgrounds within medical physics is a widely held sentiment within the field of medical physics, a well‐defined and accredited training pathway for those seeking entry into clinical medical physics is necessary. When the American Board of Radiology (ABR) first announced the 2012/2014 initiative, which was strongly supported by the AAPM due to concerns regarding the board passing rates, it restricted entry into the medical physics ABR certification pathway to candidates enrolled in CAMPEP‐accredited programs.^6^ In response, the AAPM published AAPM Report 197S^5^ and TG‐133,^4^ and CAMPEP established standards^2^ and policies^7^ for the accreditation of certificate programs. The goal of CAMPEP‐accredited certificate programs is, at a minimum, to equip alternative pathway applicants with a graduate level medical physics didactic education required by CAMPEP. As of July 2022, there are 30 accredited certificate programs.

According to CAMPEP standards^2^ and policies,^7^ students enrolling in a certificate program (i) shall have a strong background in basic physics and (ii) must hold a PhD in physics or a related discipline *prior* to matriculating into the program. In the absence of an undergraduate or graduate degree in physics, the student must have the equivalent of a minor in physics. A certificate program must address the following six core topics^5^:

1. Radiological physics and dosimetry
2. Radiation protection and radiation safety
3. Fundamentals of imaging in medicine
4. Radiobiology
5. Anatomy and physiology
6. Radiation therapy physics

Currently, certificate programs can reside in one of two settings. The certificate program may be contained within a CAMPEP‐accredited graduate program. As of July 2022, ∼83% of certificate programs (25 of 30) follow this structure. The accreditation process for such a program requires (i) a listing of the specific courses within the accredited graduate program that sufficiently address the six core topics listed earlier, and (ii) an attestation from the graduate Program Director that the courses reviewed for the accreditation of the graduate program are the same as those used for the certificate program. The second current setting is to house the certificate program within a CAMPEP‐accredited residency program that is associated with a higher education institution, even if that institution does not offer a medical physics graduate program. For this option, the accreditation process requires completion of the graduate application template by the program to demonstrate that the six core topics are addressed with detailed descriptions of the courses offered. Normally, a site visit will be required to evaluate the courses, to seek input from existing or potential students (where available), and to review teaching faculty and the educational environment. Association of a certificate program with a residency might allow, for example, an institution to admit PhD graduates from other disciplines of physics, to prepare them through the didactic certificate training, and then to consider them for their own residency training program.

Although the support of the alternative pathway through accredited certificate programs has been generally well adopted within medical physics, several concerns have arisen over the past years regarding structure and delivery of certificate programs described as follows.

Although CAMPEP standard 7.4 states that all students, including those in certificate programs, “shall have access to appropriate clinical and research facilities and the program shall demonstrate that clinical facilities and equipment are used in the teaching of practical aspects of core topics,”^2^ one may still question whether certificate students have largely the same exposure since the six core topics often do not include significant hands‐on laboratory and experiential learning. A possible consequence of this scenario is that a certificate‐prepared graduate could subsequently enter a residency training program without in‐person familiarity with imaging or radiation therapy equipment or instrumentation, for example. This situation would be particularly acute in the case of online certificate programs if the student were isolated from these resources for the duration of the program.

An example of graduate standards waived for certificate students are attendance and participation at conferences, seminars, and journal clubs to allow “students to practice their presentation and oral communication skills,” as well as a set of 24 curricular standards under the headings of professionalism, leadership, and ethics. These exceptions are, in fact, in keeping with the requirement for addressing only the six curricular areas listed earlier. With regard to professionalism and ethics topics, the CAMPEP standards^2^ indicate that although these topics should be introduced in graduate educational programs, they are anticipated to be covered in greater detail over the course of residency training.

Another potential concern relates to the timing of certificate admission and completion of the coursework. Although the original intention of the certificate program was to provide an avenue into the medical physics profession for PhD graduates from non‐medical physics specializations of physics, the expectation was—and the requirement remains—that those entering a certificate program already hold doctoral degrees. Some institutions have advocated for allowing concurrent enrollment in certificate courses with a non‐medical physics doctoral program, which is not permitted according to current CAMPEP requirements,^7^ as this could result in awarding the certificate before or coincident with a non‐CAMPEP doctoral degree.

## 3 CURRENT STATUS OF CERTIFICATE PROGRAMS/ALTERNATIVE PATHWAY CANDIDATES

**FIGURE 1:**
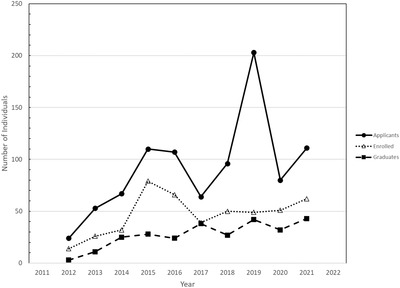
Plots and time trends in the number of applicants to certificate programs and the number of trainees enrolled and graduating from certificate programs

In 2021, there were a total of 111 applicants and 62 enrollees in certificate programs.^8^ Of those matriculated, a total of 43 completed the certificate program requirements, which is ∼39% higher than the average matriculation rate in previous years (2014–2020). A summary of the destination of graduates from certificate programs is provided in Table 1. Figure 1 illustrates trends in the number of applicants, students enrolled, and students graduating each year.

**TABLE 1:** A summary of the destination of certificate program graduates^8^

		Entered residency						
Year	No. of graduates	RT	DI	Junior MP position	Non‐MP position	Position in academia	Still seeking position	Other
2012	3	1	0	0	–	0	0	0
2013	11	7	0	0	–	0	0	1
2014	25	10	0	3	1	3	2	5
2015	28	17	1	0	0	1	2	1
2016	24	11	1	1	1	2	4	1
2017	38	12	1	3	0	4	0	5
2018	27	15	3	3	0	6	2	1
2019	42	27	4	3	1	7	2	1
2020	32	18	0	2	1	7	1	1
2021	43	25	4	5	2	7	0	0

*Note*: The table does not include data from a program that has closed to new enrollment.

Abbreviations: DI, diagnostic imaging; MP, medical physics; RT, radiation therapy.

Objective evaluation of the success of the alternative pathway is challenging but the ABR certification examination represents one potentially relevant metric. As the quality of a candidate's performance on parts 2 and 3 of the ABR exam should be largely determined by their residency training, a review of the ABR part 1 pass rates may provide insight into the relative preparedness of alternative pathway candidates for entry into residency programs, which could then be used to help drive program improvements. Pass rate data from the 2016–2022 ABR part 1 exams were compared between those who graduated from a certificate and those who did not.^9^, ^10^ (The 2018 exam was not included due to an abnormality in the exam.) For these 5 years, 72/145 (50%) certificate candidates passed the exam, whereas 551/898 (61%) non‐certificate candidates (i.e., candidates from CAMPEP‐accredited medical physics graduate programs) passed the exam. Although this difference is statistically significant (*p* = 0.008 using a *T*‐test for proportion), it is unclear whether this is associated with characteristics of the certificate pathway itself (e.g., length of time in medical physics graduate study, extent of medical physics coursework, or some other aspect of the nature of the certificate program structure) or with characteristics of individual applicants and/or programs that may have affected the overall result. Therefore, it should be clear that one cannot make inferences from these data about either the certificate pathway itself or about any individual program. We encourage individual programs to evaluate their own pass rate data and encourage CAMPEP to evaluate this aspect of program performance.

Beginning in 2019, CAMPEP included a question on the academic background (i.e., physics, engineering [nuclear, biomedical, electrical], biophysics, applied physics, chemistry, mathematics, other) of newly accepted students in their annual graduate program evaluation to help provide insight those in certificate programs. However, with several years of data, one cannot discern whether the intent of allowing the alternative pathway as expressed in Section 2 of this document is being achieved (see Tables 2 and 3). Ideally, bringing individuals into the field with a different background and knowledge base can infuse the field with new insights, ideas, and perspectives.

**TABLE 2:** Academic background of students entering graduate programs as reported by program directors in their Commission on Accreditation of Medical Physics Educational Programs (CAMPEP) annual report^8^

	Discipline								
				Engineering					
Year	Physics	Biophysics	Applied physics	Nuclear	Biomedical	Electrical	Chemistry	Mathematics	Other
2019	49	13		12	16	3		–	15
2020	51	12		12	13	9		–	–
2021	55	13	13	17	12		2	7	40

**TABLE 3:** Academic background of students entering certificate programs as reported by program directors in their Commission on Accreditation of Medical Physics Educational Programs (CAMPEP) annual report^8^

	Discipline								
				Engineering					
Year	Physics	Biophysics	Applied physics	Nuclear	Biomedical	Electrical	Chemistry	Mathematics	Other
2019	24	3		6	7	2			5
2020	17	5		2	6	3			11
2021	17	4	3	2	7	3	1	1	7

## 4 SUMMARY

Based on the feedback provided by residency program directors and others associated with the education of medical physicists, this TG was asked to address several concerns that arose after the writing of AAPM Report 197S. The concerns are delineated in Sections 4.1–4.6. The TG deliberated on these concerns and proposes the following recommendations.

### 4.1 Determine whether the educational requirements stated in AAPM Report 197S were sufficient or whether there were educational components that need to be added

*Task Group Recommendation*. Certificate programs shall include professionalism, leadership, and ethics as a component within their core curriculum as specified in AAPM Report 365.^11^ Further review of the didactic requirements for alternative pathway candidates are currently underway by the Working Group on Medical Physics Graduate Education Program Curriculum and are expected to be included in AAPM Report 365.

### 4.2 Assure that certificate programs are delivered within a suitable academic environment with appropriate academic resources

*Task Group Recommendation*. We recommend that certificate programs should reside within a CAMPEP‐accredited graduate program. Certificate programs may also reside in an institution with a CAMPEP‐accredited residency program, provided there is a strong affiliation with and support from an accredited college or university, and that the program instructors have faculty appointments within that academic institution.

### 4.3 Address a perceived lack of clinical exposure and professional understanding during the training of certificate program students

*Task Group Recommendation*. Certificate programs shall include exposure to clinical applications of didactic material within the core curriculum as specified in Section 2 of this report.

### 4.4 Address concerns with online delivery of certificate program content

*Task Group Recommendation*. Online delivery of core curriculum material should follow the guidelines of the accredited college or university, but the type and amount of material offered online should be judiciously evaluated. A minimum level of in‐person instruction (>0%) is recommended to assure the certificate student receives exposure to experiential, clinical medical physics.

### 4.5 Address the issue of a student concurrently pursuing the completion of a non‐CAMPEP‐accredited PhD and a certificate program

*Task Group Recommendation*. We recommend that students enrolled in a certificate program should have a terminal PhD degree in physics or related field, consistent with the CAMPEP standards and policies as of the submission of this report (July 2022). Moreover, it is not acceptable for a student to be enrolled in a non‐medical physics PhD and enrolled in a certificate program at the same time.^2^ Training in medical physics requires focus on medical physics content and should not be mixed with and potentially distracted by non‐medical physics training. Further, a pathway allowing concurrent enrollment would undermine medical physics graduate programs and those students that have pursued the medical physics Masters and/or Doctoral degree(s). Additionally, allowing concurrent enrollment would circumvent the current alternative pathway and the rationale for this pathway. Therefore, we recommend that concurrent enrollment in a non‐CAMPEP PhD program and a medical physics certificate program should not be allowed.

### 4.6 Assess whether certificate programs are meeting their goal of adequately preparing individuals for entry into medical physics residency programs

*Task Group Recommendation*. We recommend follow‐up investigations and surveys to determine the success of the certificate program initiative. As the certificate program is explicitly designed to provide a mechanism for preparation for an accredited residency training program, evaluations of such programs should include, at a minimum, the success rate in placing graduates into these positions. Additional evaluations should include an assessment of the certificate program's success in meeting the stated goals of their program. Programs should request ABR part 1 data for their certificate and graduate students and residents for comparison and evaluation of trends. Consistent with CAMPEP's graduate standards, we recommend programs post the subsequent positions of their graduates (e.g., residency and industry) on their program's website for transparency.^2^

Although we cannot currently comment on whether the alternative pathway approach will attract the best possible scientists to our field, or whether we need to adjust the number of certificate programs, we hope these questions can be addressed in the future by continuing to monitor and evaluate alternative pathway programs and the successes/accomplishments of their graduates (e.g., publications, patents, and grants). Further, we acknowledge that there may be other solutions that address the concerns discussed in this report, which may result in changes in future recommendations of certificate program/alternative pathway candidate education and training.

## AUTHOR CONTRIBUTIONS

All authors contributed to developing the recommendations detailed in this report, as well as writing the report. As the Task Group (TG) chair, Dr. Seibert organized and led the TG meetings, as well at the structure of the report. The preamble was developed by Drs. Burmeister and Robar. Dr. Prisciandaro maintained the running draft of the report and led efforts in the editing of the report.

## CONFLICT OF INTEREST

The authors declare they have no conflicts of interest.
